# A Beam Orientation Quality Assurance Framework for Gyroscopic Stereotactic Radiosurgery Systems

**DOI:** 10.7759/cureus.88922

**Published:** 2025-07-28

**Authors:** Alec Zheng, Timothy Kong, Ajay Zheng, Xiang Kong, Xiaodong Wu, Aizik Wolf

**Affiliations:** 1 Miami Neuroscience Center, Larkin Community Hospital, Miami, USA; 2 Neuroscience and Behavioral Biology, Emory University, Atlanta, USA; 3 Arts and Sciences, University of Pennsylvania, Philadelphia, USA; 4 Medical Physics, New York Proton Center, New York, USA; 5 Medical Physics, Executive Medical Physics Associates, Miami, USA

**Keywords:** angular deviation, beam orientation, diode array, quality assurance (qa), zap gyroscopic radiosurgery

## Abstract

Gyroscopic stereotactic radiosurgery (SRS) systems, such as ZAP-X, enable precise, isocentric beam delivery with high conformality and minimal exposure to surrounding tissues. While standard quality assurance (QA) protocols verify beam centricity and overall targeting accuracy, they are not specifically designed to assess the consistency of beam entry angles, which can vary in systems employing gimbaled delivery. These angular variations, though often less consequential at the target, may have significant dosimetric implications for peripheral critical structures. To address this, we developed a practical quality assurance framework using the ArcCHECK cylindrical diode array for periodic beam orientation monitoring. The system comprises 1,386 diodes arranged cylindrically (21 cm diameter), providing a density of 221 diodes per 10×10 cm². Although the array’s resolution limits direct dose distribution comparisons, its sensitivity to angular deviations leverages the steep dose fall-off typical in SRS beams. A calibration protocol was established using a 44-beam QA plan with collimators of varying sizes. Sensitivity to beam rotation was quantified as the percentage of diode failures per degree of angular deviation. The 10 mm collimator demonstrated optimal performance, with sensitivity reaching 37.7% per degree, owing to the match between its penumbra and the 1 cm diode spacing. Daily QA measurements confirmed high reproducibility and sensitivity, with a deliberately introduced 0.8° error accurately detected and quantified. This framework, validated through controlled tests, offers a reliable and efficient method to track beam orientation consistency without requiring additional hardware or major workflow changes. While the study serves as a proof of principle within a single institution, broader adoption and multicenter validation could support its standardization. Ultimately, the integration of angular QA enhances treatment fidelity in gyroscopic SRS, ensuring both mechanical precision and patient safety.

## Introduction

Gyroscopic stereotactic radiosurgery (SRS) systems, such as ZAP-X, enable highly conformal, isocentric delivery for the treatment of intracranial and cervical lesions [1]. The dual-axis gyroscopic design allows the beam to approach the target from a wide range of solid angles, increasing conformity and minimizing dose to surrounding tissues [1,2].

To assure consistent performance, a set of quality assurance (QA) procedures has been developed and recommended by the vendor [1], in close accordance with the AAPM Task Group (TG) 101 [3] and TG135 [4]. In particular, the QA for individual beam iso-centricity is carried out by a procedure similar to the Winston-Lutz test, and the overall targeting accuracy is performed by the end-to-end (E2E) test with the humanoid RANDO Phantom.

For an isocentric delivery from multiple beams, the total targeting accuracy indicated by the E2E test is not sensitive to the beam entry angles. The Winston-Lutz test only evaluates beam centricity rather than the beam entry angle. Unlike Gammaknife, where angular entries of all beams are mechanically fixed, ZAP-X adjusts each beam’s entry angle sequentially to the pre-programmed position using dual-gantry gimbals. While the gimbal configuration ensures reliable beam centricity, the entry angles may vary.

From a dosimetric perspective, SRS treatment delivered iso-centrically by the ZAP-X is highly reliable near the target area in terms of beam entry angles (orientation). However, for critical organs located relatively far from the target, variations or errors in beam entry angle can be significant. This necessitates periodic QA to ensure the consistency of beam orientation with the ZAP-X, alongside commonly adopted routine QA protocols [3-5].

Here, we present a practical, sensitive QA framework using the ArcCHECK cylindrical diode array (Sun Nuclear Corp., Melbourne, FL) for periodic monitoring of beam orientation, integrated into the clinical workflow for ZAP-X or similar gyroscopic SRS platforms.

The ArcCHECK system is widely adopted for patient-specific QA in modern radiotherapy. Its cylindrical design and helical diode array, with rotational invariance, enable reliable assessment of 3D dose measurement from complex deliveries, including volumetric modulated arc therapy (VMAT) and intensity modulated radiation therapy (IMRT), as well as SRS and stereotactic body radiotherapy (SBRT) [6-10]. Traditionally, ArcCHECK uses gamma analysis for 3D dose analysis. Although studies confirmed its high gamma passing rates (>95%) at 3%/3 mm for stereotactic plans with linac-based SRS, unlike film QA, its sensitivity decreases with stricter criteria such as 2%/1 mm, indicating the unfavorable influence of detector spacing and resolution [6,7,10]. Compared with high-resolution devices and film, ArcCHECK may be less sensitive to small apparatus errors but remains effective when proper gamma indices are chosen for larger targets (e.g., 2%/3 mm for SRS, or 2%/2 mm for SBRT) [7,8,11,12]. Collectively, these attributes establish ArcCHECK as an efficient platform for SRS/SBRT QA, provided its physical and algorithmic limitations are recognized and addressed.

Our developed framework extends the application of ArcCHECK beyond its standard gamma analysis protocol, demonstrating its distinctive capability to detect variance in beam orientation with the ZAP-X system.

## Technical report

Principle of ArcCHECK-based angular error detection

The principle behind using the ArcCHECK cylindrical diode array for beam orientation QA lies in its ability to exploit the sharp dose gradients characteristic of SRS beams, even with a relatively modest detector density.

Unlike high-resolution film or 2D arrays, ArcCHECK features 1,386 n-Si diodes (0.019 mm³ each) distributed helically along the inner wall of a 21 cm diameter acrylic cylinder, with 1 cm spacing between adjacent detectors, yielding a density of 221 diodes per 10 × 10 cm² area [13]. Although this spacing may be considered sparse for fine spatial dosimetry, the method’s effectiveness arises from the unique interplay between the beam’s geometric properties and the physical arrangement of the diodes.

**Figure 1:**
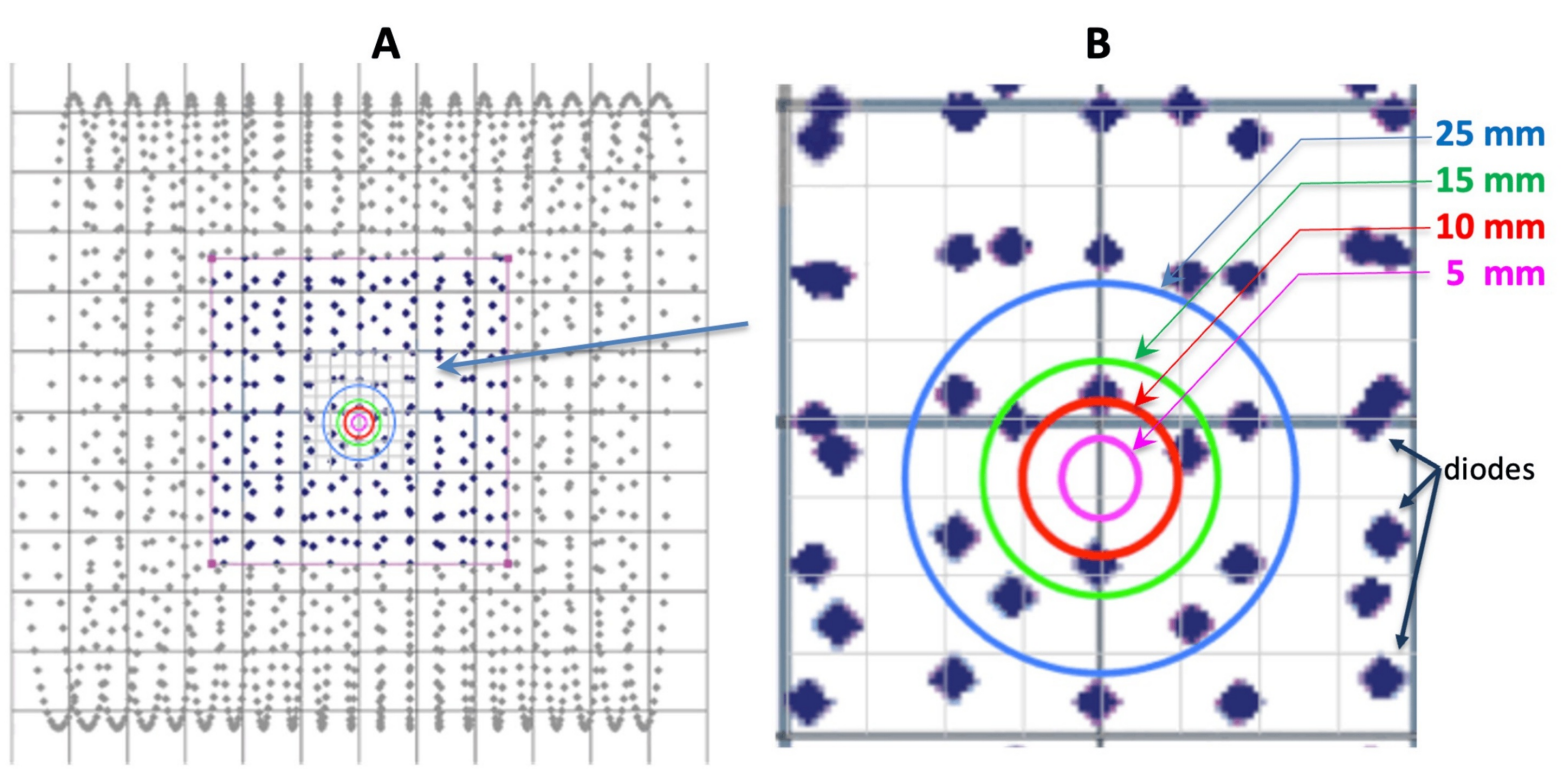
The layout of spiral diodes within the ArcCHECK system (A) and their alignment with the beams from collimator cones of 5 mm, 10 mm, 15 mm, and 25 mm (B) (zoom-in view).

SRS beams, particularly those collimated to small diameters (e.g., 5-25 mm), produce extremely steep dose gradients at the beam edge. When the beam passes through the ArcCHECK cylinder, even a slight angular misalignment results in a measurable shift in the pattern of diode response due to the abrupt change from high to low dose at the edge of the beam. As the entry angle of the beam varies - even by tenths of a degree - the region of steep dose fall-off traverses a different subset of diodes, resulting in marked changes in the measured signal. Figure 1 illustrates the diode distribution and the projected cross sections of beams with 5-25 mm collimators.

**Figure 2:**
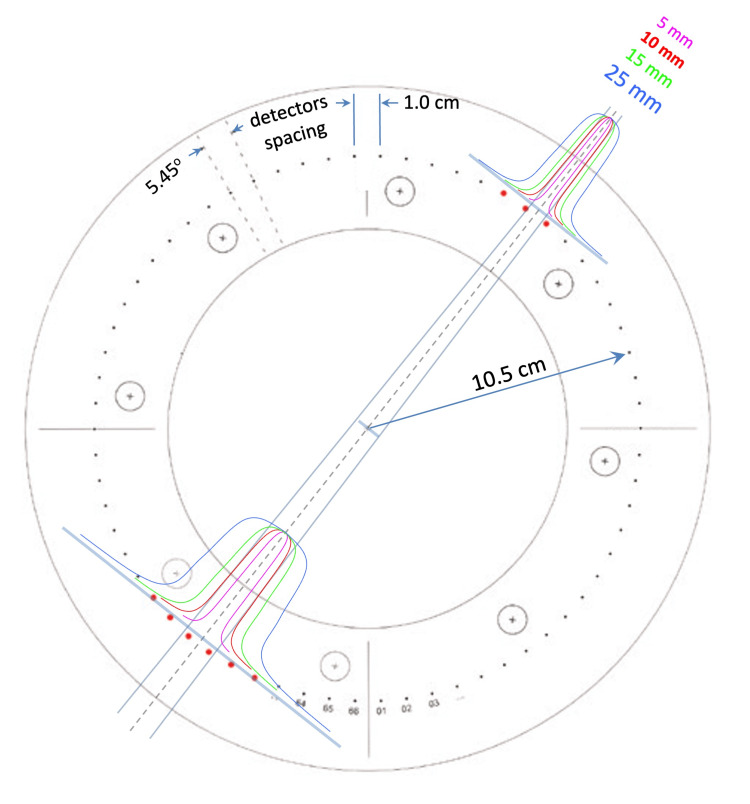
The geometric separation of the ArcCHECK diodes relative to lateral beam profiles from collimator cones of 5 mm, 10 mm, 15 mm, and 25 mm.

This “gradient amplification” effect means that the sensitivity of the system to angular variance is determined less by the absolute detector density and more by the steepness of the dose profile. For instance, with a 10 mm collimator, the spatial extent of the dose penumbra matches well with the 1 cm diode spacing, allowing for optimal sensitivity to angular deviation. Thus, while ArcCHECK may not be capable of reconstructing fine details of planar dose distributions, it excels at detecting systematic deviations in beam angle, as these produce large, easily quantifiable changes in the set of diodes registering above-threshold signals. Figure 2 illustrates the beam profiles of collimators ranging from 5 mm to 25 mm, highlighting the sharp gradients of the penumbra as they move across the detectors.

The QA protocol leverages the comparison of measured diode response patterns to a baseline acquired under ideal alignment. Any shift in the angular orientation of the delivery system manifests as a change in the relative signal intensities across the array. Instead of using traditional gamma analysis protocols, the detection metric is defined as the increase in percent of failing diodes in, dose only, per degree deviation. The relationship between the percent of failing diodes and the degree of rotation is empirically determined, enabling the system to subsequently monitor the beam orientation consistency over time.

QA procedure for beam orientation

The proposed QA procedure for monitoring beam orientation in gyroscopic SRS systems using ArcCHECK is designed to provide both routine verification and quantitative calibration of system sensitivity to angular deviations. The workflow is structured to be practical, repeatable, and robust against user or setup variability. Below, the procedure is described stepwise, with sensitivity calibration included as an integral component.

QA Plan Generation

A dedicated QA treatment plan is developed, consisting of multiple narrow beams with a single isocenter and utilizing various collimator sizes. The isocenter is precisely positioned at the geometric center of the ArcCHECK cylinder, ensuring that any beam rotation will alter the pattern of diode activation. Plans should cover the clinically relevant range of beam entry angles.

Baseline Measurement Acquisition

Following comprehensive system tuning (annual QA or major maintenance service), under precise image-guided alignment, the initial QA plan is delivered to the ArcCHECK device. The resulting dose matrix, as recorded by the diode array, is stored in the QA software database and serves as the baseline reference for future measurements.

Sensitivity Calibration (Angular Sensitivity Analysis)

To quantify and calibrate the system’s ability to detect angular deviations, additional QA plans are generated with known, controlled rotational offsets (e.g., 0.5°, 1.0°, 2.0°, and 3.0°). These are delivered sequentially to the ArcCHECK under otherwise identical conditions. For each induced rotation, the measured diode response is compared to the baseline using specified testing criteria (e.g., 3% dose difference and 10% threshold). The percent of diodes failing the comparison is plotted as a function of rotational deviation, yielding a calibration curve (sensitivity function) that quantifies how failure rate scales with angular error for each collimator size. This calibration is essential for interpreting routine QA results in absolute angular terms.

Routine QA Delivery

For routine QA, the baseline device reading should be obtained immediately after commissioning or following annual QA. At defined intervals (daily, weekly, or monthly), the same QA plan is re-delivered under standard setup. The measured dose distribution is then compared against the baseline to obtain the failure rate using the same criteria established during the initial sensitivity calibration.

Interpretation and Thresholding

Any increase in the percentage of failing diodes relative to the baseline is interpreted using the previously established calibration curve. This allows direct estimation of the magnitude of any angular deviation, with the system’s detection limit defined by the slope on the calibration curve.

Action and Documentation

If the estimated angular deviation exceeds a pre-defined clinical threshold, the source of misalignment is investigated and corrective action taken. All results are documented as part of the ongoing QA program.

Key features and advantages

With gradient amplification, the use of sharply collimated beams and the cylindrical geometry of the ArcCHECK means that even small angular shifts produce large, easily detectable changes in the diode activation pattern. For certain collimator sizes, most notably 10 mm, the spacing of the diode array closely matches the beam’s penumbra, resulting in an optimal match of spatial resolution and maximizing sensitivity to rotational drift. Empirical sensitivity calibration further ensures that observed QA deviations can be reliably interpreted as actual angular errors, rather than relying solely on relative changes.

This integrated QA procedure ensures both immediate detection of beam orientation errors and provides a quantitative basis for tracking mechanical integrity and alignment consistency of gyroscopic radiosurgery systems over time.

Results

Sensitivity Calibration and Collimator Optimization

**Figure 3:**
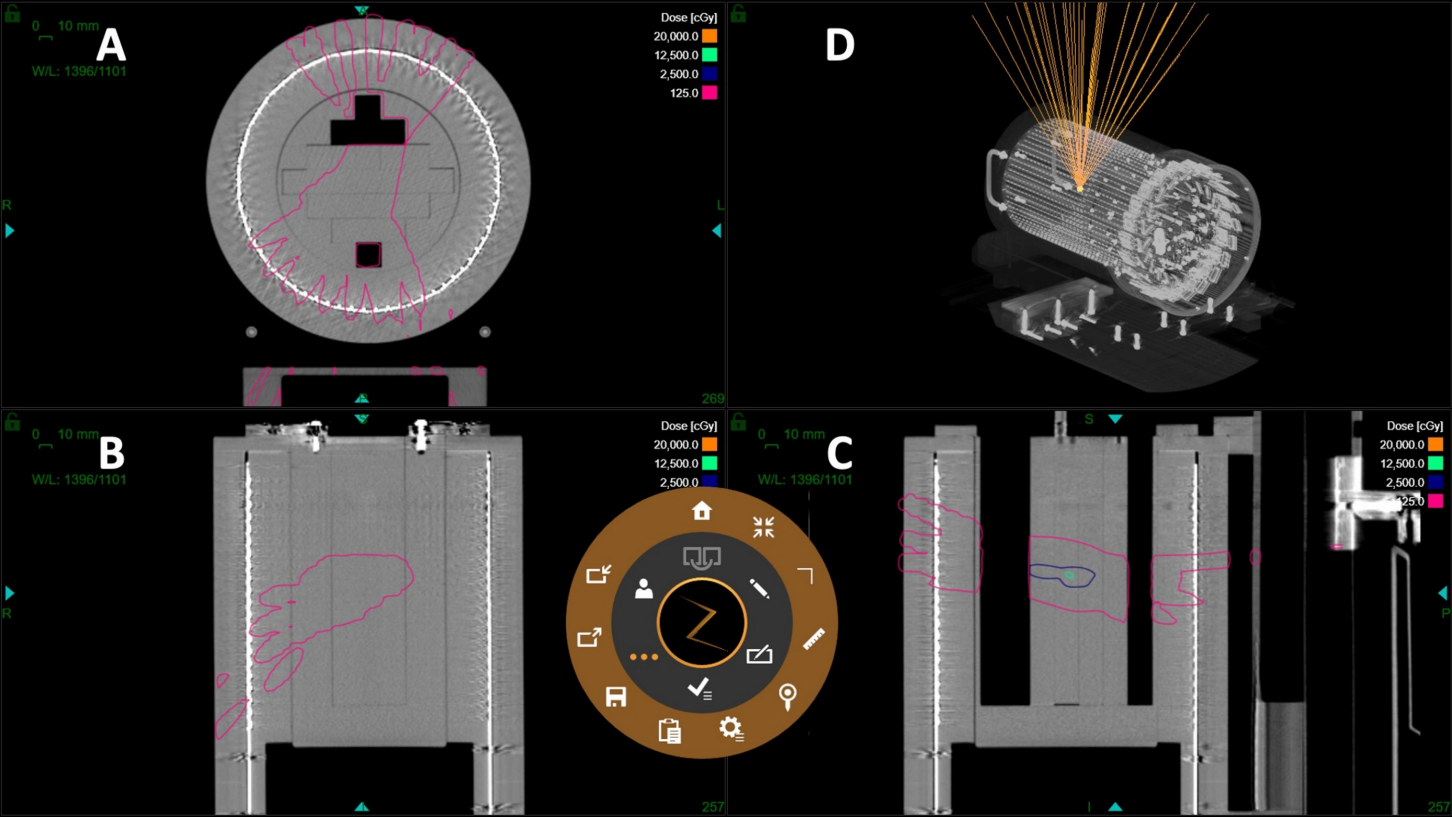
Design of the QA plan. Schematic illustrating the 44-beam QA plan converging at a single isocenter within the ArcCHECK cylindrical diode array. Various collimator sizes (5 mm, 10 mm, 15 mm, 25 mm) are used to assess angular sensitivity. A, B, and C: Axial, coronal, and sagittal views of the QA plan, respectively. D: 3D trajectories for all 44 beams.

The sensitivity calibration phase was critical in establishing the capability of the ArcCHECK system to detect angular deviations and in identifying the optimal collimator size for routine QA. To this end, a dedicated QA plan was designed, consisting of 44 non-coplanar beams converging at a single isocenter, each delivered through cones of various diameters: 5 mm, 10 mm, 15 mm, and 25 mm (Figure 3).

**Figure 4:**
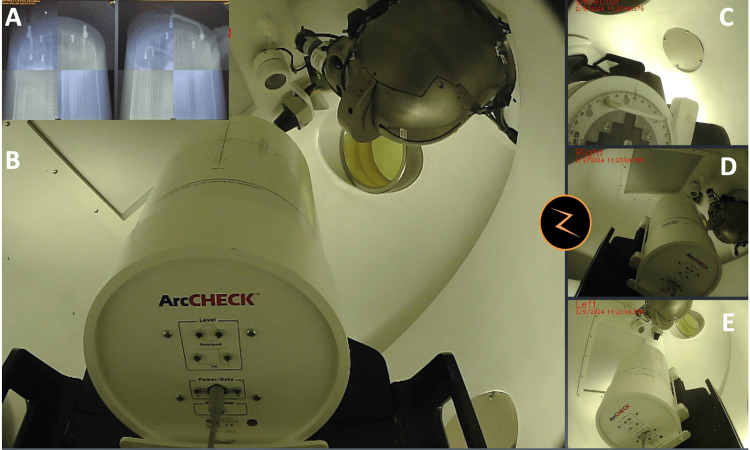
Measurement setup. The ArcCHECK device positioned at the isocenter of the ZAP-X system (B-E) under stereotactic image-guided alignment (A), demonstrating the practical setup for beam orientation QA.

These plans were delivered to the ArcCHECK system, which was positioned at the ZAP-X isocenter and aligned under image guidance to ensure reproducibility and eliminate setup bias (Figure 4).

**Figure 5:**
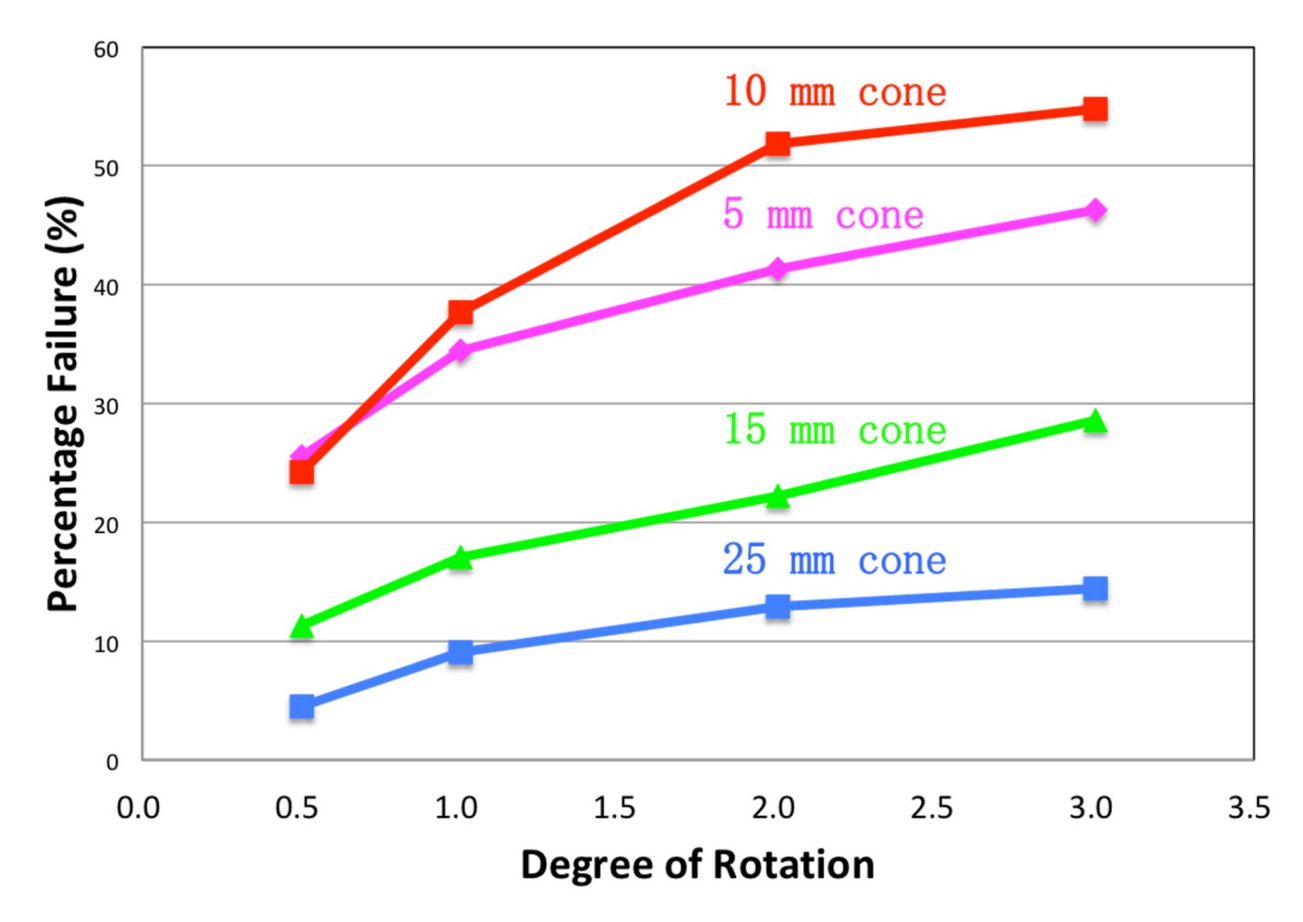
Sensitivity calibration curves. Plots of percent diode failure versus introduced rotational deviation for each collimator size. The 10 mm collimator exhibits the highest sensitivity, with the steepest increase in diode failure rate per degree of rotation.

For each collimator, baseline measurements were acquired. Next, additional QA plans were delivered with deliberate angular deviations of 0.5°, 1.0°, 2.0°, and 3.0°, allowing the construction of sensitivity calibration curves. The percentage of diode failures (using a 3% dose difference and 10% threshold) was plotted against the introduced angular deviation for each collimator size (Figure 5).

Analysis of the curves revealed that the 10 mm collimator provided the greatest sensitivity to beam rotation, with a response of 37.7% failing diodes per degree. This performance is attributed to the close match between the spatial beam profile at 10 mm and the 1 cm detector spacing of ArcCHECK. In comparison, the 5 mm cone showed low sensitivity due to the minimal number of intersected diodes, while larger cones (15 mm and 25 mm) demonstrated a decrease in sensitivity as the broader beam profile reduced the gradient at the beam edge. Accordingly, the 10 mm collimator was selected as the optimal configuration for ongoing routine QA.

Routine QA - Daily Measurement Analysis

Following baseline establishment and calibration, eight consecutive daily QA measurements were performed using the 10 mm collimator configuration. The same QA plan was delivered each day under image-guided alignment. On the third day, a deliberate rotational error of 0.8° was introduced, while standard alignment was used on all other days. The percentage of dose failure was used to determine the degree of beam rotation according to the pre-established calibration curve. These calculated rotation values were then compared to those obtained from the auto-alignment procedure, which relies on the structural features of the ArcCHECK device.

**Figure 6:**
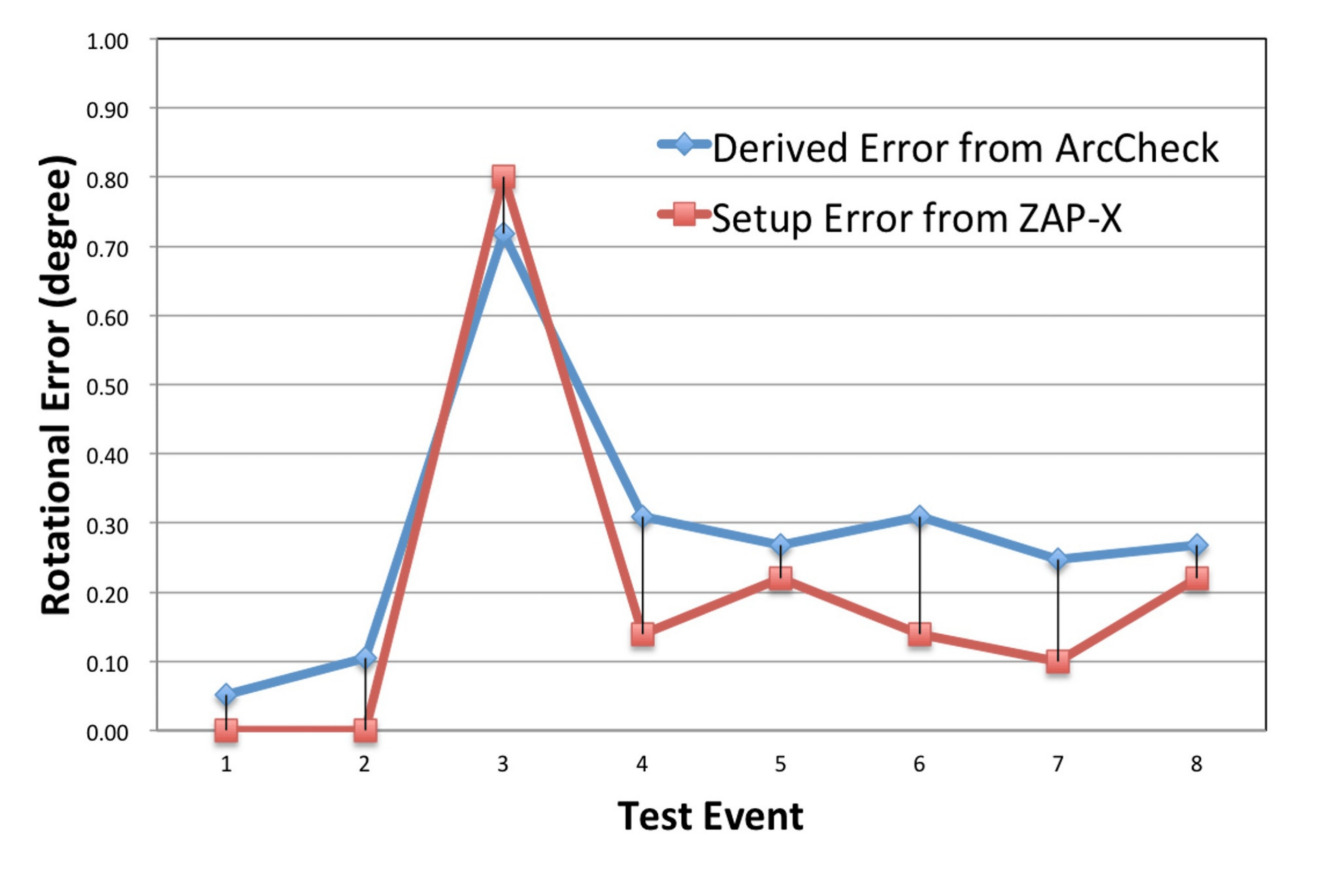
Eight-day quality assurance (QA) results. Beam rotational variance from eight consecutive daily QA measurements using the 10 mm collimator: Stable readings were observed on seven out of the eight days, while an intentional 0.8° rotation introduced on day three resulted in a significant increase in diode failure rate as expected. The calculated angular variations well align with the values reported by the ZAP-X image-guiding system.

Analysis showed that the first two measurements and days four through eight exhibited consistently low diode failure rates, confirming the mechanical stability and reproducibility of the ZAP-X system. On day three, as expected from the sensitivity calibration, the induced 0.8° rotational error resulted in a marked increase in the failure rate, closely matching the calibration curve prediction. The ArcCHECK-derived measurement of rotational error agreed with the system-reported value within 0.10 ± 0.05°, confirming both sensitivity and quantitative reliability (Figure 6).

## Discussion

Although current QA protocols for ZAP-X are well suited to ensure the system’s iso-centricity, a noticeable gap in QA pertaining to beam entry angles is evident [1,4,5]. This study demonstrates a novel, practical approach for systematic beam orientation quality assurance in gyroscopic SRS systems using the ArcCHECK cylindrical diode array. The results provide proof-of-principle that, even with relatively modest detector density, ArcCHECK can sensitively and reproducibly detect small angular deviations in beam entry, particularly when leveraging the steep dose gradients and geometric features of stereotactic beams.

The sensitivity calibration phase established that the 10 mm collimator offers optimal detection characteristics, as its projected beam width aligns closely with the 1 cm diode spacing of the ArcCHECK array [13]. This synergy results in a pronounced response to angular deviations - quantified at nearly 38% diode failure per degree - while smaller or larger collimators were limited by undersampling or insufficient gradient, respectively. Routine daily QA, further validated by deliberate introduction of a 0.8° error, showed that this method can not only detect but also quantify rotational deviations, with excellent agreement between ArcCHECK-derived and imaging-reported errors.

Interpretation of these findings underscores a key strength of the method: rather than depending on high-resolution mapping of the entire dose distribution, this QA approach capitalizes on the physical properties of SRS delivery and the relative response of the diode array to sharp beam gradients. For clinical practice, this means reliable, rapid detection of angular errors that could otherwise compromise the safety of adjacent critical structures, even if the impact at the target center is minimal.

However, it is important to recognize that this work primarily serves as a proof-of-concept. While the results were robust within our single-institution dataset, the broader clinical utility and generalizability of this QA protocol will require further validation. Presented at the 2024 Radiosurgery Society (RSS) Annual Meeting [14], this work serves as a foundation in the hope that a broader consensus on its merits and feasibility can be established, paving the way for valuable multicenter collaboration. The accumulation of a larger, cumulative QA dataset will be essential to fully assess the robustness, reproducibility, and generalizability of this framework across different machines, workflows, and clinical environments. Real-world variations in setup technique, imaging accuracy, and equipment-specific factors may affect sensitivity and operational thresholds, emphasizing the need for comprehensive multicenter validation.

Nevertheless, this study provides a solid foundation and a practical methodology for routine beam orientation QA in gyroscopic SRS systems. The framework is readily adaptable and efficient and does not require additional hardware or extensive training beyond the standard ArcCHECK operation.

## Conclusions

We have established and validated a proof-of-principle protocol for beam orientation QA in gyroscopic SRS, utilizing the ArcCHECK cylindrical diode array. Sensitivity calibration confirms that the 10 mm collimator configuration optimally matches the physical and dosimetric characteristics required for precise angular detection. The approach is capable of detecting and quantifying small rotational errors with high sensitivity, offering a practical means to maintain and document the mechanical integrity of SRS delivery systems.

Moving forward, the routine application of this QA framework, combined with the accumulation of additional data from repeated clinical use and from other institutions, will be crucial to further verify and refine its performance. A multicenter collaborative approach would greatly enhance our understanding of the method’s robustness and pave the way toward establishing standardized beam orientation QA protocols for the broader radiosurgery community.
